# High-flux soft x-ray harmonic generation from ionization-shaped few-cycle laser pulses

**DOI:** 10.1126/sciadv.aar3761

**Published:** 2018-05-11

**Authors:** Allan S. Johnson, Dane R. Austin, David A. Wood, Christian Brahms, Andrew Gregory, Konstantin B. Holzner, Sebastian Jarosch, Esben W. Larsen, Susan Parker, Christian S. Strüber, Peng Ye, John W. G. Tisch, Jon P. Marangos

**Affiliations:** Blackett Laboratory, Imperial College London, Prince Consort Road, London SW7 2AZ, UK.

## Abstract

Laser-driven high-harmonic generation provides the only demonstrated route to generating stable, tabletop attosecond x-ray pulses but has low flux compared to other x-ray technologies. We show that high-harmonic generation can produce higher photon energies and flux by using higher laser intensities than are typical, strongly ionizing the medium and creating plasma that reshapes the driving laser field. We obtain high harmonics capable of supporting attosecond pulses up to photon energies of 600 eV and a photon flux inside the water window (284 to 540 eV) 10 times higher than previous attosecond sources. We demonstrate that operating in this regime is key for attosecond pulse generation in the x-ray range and will become increasingly important as harmonic generation moves to fields that drive even longer wavelengths.

## Teaser

X-ray harmonics made with long-wavelength lasers reach higher fluxes and photon energies when plasma effects dominate.

## INTRODUCTION

The generation of attosecond pulses in the extreme ultraviolet spectral region (20 to 150 eV) has enabled time-resolved measurements of attosecond dynamics in a variety of systems, including atoms (*1*), molecules (*2*), and solids (*3*). The advantages of high-harmonic-generation (HHG)–based sources include the potential for subfemtosecond pulse duration, intrinsic synchronization to the driving field, and relative compactness. In parallel, the capability of x-ray sources to address specific atomic absorption edges and to image nanoscale structures has motivated the development of large-scale light sources such as time-sliced synchrotron beamlines (*4*) and free-electron lasers (*5*). The unification of HHG-based attosecond pulses with x-ray spectroscopy and imaging techniques, particularly for time-resolved x-ray absorption spectroscopy (TR-XAS) (*4*, *6*, *7*), has stimulated research into soft x-ray (SXR) HHG for more than two decades (*8*, *9*). Recent advances include carrier-envelope-phase (CEP)–dependent high harmonics across the carbon K-edge at 284 eV and up to the oxygen K-edge at 540 eV (*10*–*15*), measurement of attosecond pulses in and near the water window (284 to 540 eV) (*16*–*18*), and the first demonstrations of TR-XAS with HHG sources (*7*, *19*). Despite these advances, the generation of attosecond pulses in the water window and beyond remains challenging and poorly understood. Photon fluxes are limited to around 10^8^ photons/s (integrated across the water window) (*14*, *16*, *20*), and simplified models of the macroscopic buildup offer no consensus on routes to increased efficiency (*9*, *14*).

Here, we present a new route toward the optimal generation of bright SXR attosecond pulses. We show both experimentally and theoretically that strongly overdriving the generation medium—creating a plasma through high ionization, which then spatiotemporally reshapes the driving laser—results in a brighter harmonic emission with higher photon energy. This contrasts with the conventional weak-plasma limit of HHG, in which the laser can propagate as an unmodified or weakly perturbed beam over an extended distance (*9*, *21*), as well as with previous studies of SXR harmonic generation that have neglected the spatial degree of freedom (*11*, *14*). By focusing high-energy, few-cycle laser pulses at 1.8-μm wavelength into a high-pressure (0 to 10 bar) gas target at intensities much higher than the critical intensity for plasma defocusing, we generate harmonics across the water window and beyond the oxygen K-edge, with a photon flux integrated across the water window an order of magnitude higher than previous attosecond sources. We demonstrate complete tunability of the harmonic cutoff energy from 280 to 600 eV and, by measuring the CEP dependence, show that the harmonics support the generation of isolated attosecond pulses (*11*, *15*, *16*, *22*, *23*). This cutoff is almost 200 eV beyond the conventional limit for HHG with 1.8-μm driving sources (*9*, *20*). Interferometric measurements of the ionization fraction of the gas, together with comprehensive numerical modeling, show that the dynamics are dominated by the plasma response, where strong ionization-induced defocusing leads to a rapid spatiotemporal reshaping of the driving pulse, clamping the peak intensity and thus the harmonic cutoff energy, and limiting the maximum ionization fraction to just a few percent. HHG can thus be said to be in the overdriven regime when plasma lensing causes the beam to change shape rapidly, in the vicinity of high ionization and on a length scale much shorter than the Rayleigh length. Previous work on SXR harmonic generation has not considered plasma defocusing (*11*, *14*). The rapid reshaping leads to strong gradients in intensity and phase, both in space and time, and to transient subcycle phase matching. This causes the harmonics to build up rapidly as isolated attosecond bursts within a few hundred micrometers, a spatial extent much shorter than previous models have predicted. Measurements of the spatial phase of the harmonic radiation show a predominantly quadratic wavefront, demonstrating that a high-flux, high-quality source can be produced even in the overdriven limit, provided that the driving laser has minimal spatiotemporal coupling. Wavelength scaling of the plasma response and the single-atom high-harmonic emission show that, as harmonic generation moves to even higher photon energies driven by longer-wavelength lasers, the strongly overdriven route will result in even higher gains relative to the usual regime for HHG. Our high-flux, CEP-dependent, water-window harmonics constitute an ideal source for TR-XAS and x-ray microscopy measurements, and our findings help formulate a roadmap for the development of future attosecond x-ray sources.

## RESULTS

### Overdriven SXR generation

**Fig. 1:**
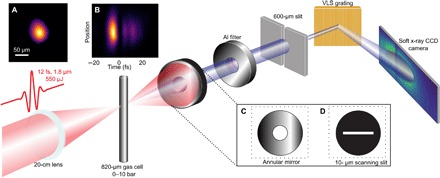
Generation and characterization of water window harmonics. Pulses of 1.8-μm wavelength, 12-fs duration, and 550-μJ energy are focused into an 820-μm outer diameter needle filled with multiple-atmosphere pressures of gas, with a spot size of 40-μm FWHM (**A**) and good spatiotemporal quality (**B**). Differential pumping keeps the chamber pressure below 10^−2^ mbar. The harmonics pass through optional diagnostic optics: a 45° annular mirror (**C**) for reflecting the IR pulses for analysis or a movable slit (**D**) for selecting a slice of the harmonics for spatial phase measurement. The harmonics then pass through metallic filters and a spectrometer slit before being detected with a flat-field grating and photon-counting x-ray charge-coupled device (CCD) camera. VLS, variable line spacing.

Figure 1 summarizes the experimental setup. We generate harmonics from CEP-stable 12-fs, 550-μJ pulses at a central wavelength of 1.8 μm and a 1-kHz repetition rate, produced using hollow-core fiber pulse compression (*15*, *24*). The beam is focused to a spot size of 40-μm full width at half maximum (FWHM) (Fig. 1A), and the nominal (that is, inferred from pulse duration, energy, and focal spot size) on-target intensity of the driving fundamental in a vacuum is more than 2 × 10^15^ W/cm^2^. This is much higher than the critical intensity for filamentation at atmospheric pressures. The excellent spatiotemporal quality of the driving pulse suppresses beam breakup, as evidenced by the symmetric and smooth spatiotemporal profile (Fig. 1B) measured using SEA-F-SPIDER (*24*). We observe no evidence of filamentation at these intensities (fig. S1), suggesting that, contrary to a recent suggestion (*19*), filamentation does not play an important role in efficient SXR harmonic generation in this parameter range.

**Fig. 2:**
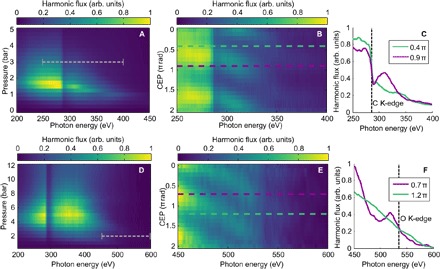
Tunable CEP-dependent harmonic generation across the water window. (**A** to **F**) Pressure dependence (A and D), CEP dependence (B and E), and spectra at two phases separated by **π**/2 (C and F) of harmonics emitted in neon (A to C) and helium (D to F). The pressure-dependent plots are shown averaged over all values of CEP. The CEP dependence is shown at the carbon and oxygen K-edges, using spectra generated at pressures indicated by the gray dashed lines in (A) and (D). Colored dashed lines correspond to the lineouts shown in (C) and (F), whereas the black dashed lines indicate the carbon and oxygen K-edges, respectively. All fluxes are shown in arbitrary units.

Figure 2 summarizes the generation results from neon (top) and helium (bottom). Figure 2 (A and D) shows the harmonic spectra as a function of backing pressure. We can optimize the flux both overall and at any particular wavelength. The highest measured total flux across the water window was 7.7 ± 2.0 pJ per pulse [(1.4 ± 0.4) × 10^8^ photons/s] in helium and 71 ± 18 pJ per pulse [(1.4 ± 0.4) × 10^9^ photons/s] in neon, a flux and conversion efficiency one order of magnitude higher than previous attosecond sources (*14*), and with a full-angle divergence below 2 mrad. Previous work using hollow capillaries has reached higher conversion efficiencies but did not demonstrate isolated attosecond pulse generation (*20*). The flux at various photon energies is summarized in table S1. Scans of the focal position relative to the gas target show that the flux is optimized when the fundamental is focused approximately 1 mm after the center of the needle (fig. S2) and that this focal position simultaneously optimizes the flux at all photon energies. This is opposite to the usual predictions made by assuming that the plasma has little influence on the driving laser (*21*). By switching gas species and adjusting the backing pressure, the harmonic cutoff can be moved to any spectral region in the water window and out to a maximum of 600 eV, providing a spectrally tunable source of x-ray radiation.

Both the oxygen and carbon K-edges are visible in the spectra because of contamination of the x-ray optics by organic molecules. Despite this, the harmonics are sufficiently bright and stable to allow for an oxygen K-edge x-ray absorption near-edge structure (XANES) measurement in a 1-μm-thick film of biaxially oriented polyethylene terephthalate (fig. S3), the highest photon energy XANES measurement made with a source of attosecond pulses to date (*25*).

**Fig. 3:**
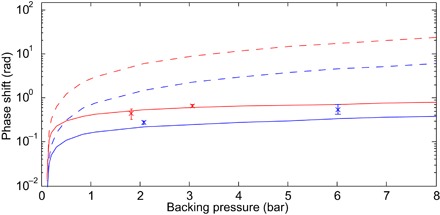
Ionization-induced phase shift (log scale) as a function of backing pressure in the target for helium (blue) and neon (red). The vacuum focus is 1.4 mm behind the gas cell; experimental data (*x*) are compared to predictions from the full-dimensional model (solid lines) and neglecting the effect of plasma upon the propagation (dashed lines). Error bars reflect the extreme values obtained over three measurements.

To verify the critical role that plasma formation plays here, we directly measured the ionization fraction of the gas target in situ via spectral interferometry while generating harmonics across the water window. Figure 3 shows the plasma-induced phase shift measured in helium and neon at various backing pressures, along with the predicted phase shift calculated using a full-dimensional model including defocusing effects (see details in section S8), and calculations where the effect of the plasma on the driving laser has been neglected, similar to previous models that ignore plasma defocusing. The experimental values are in agreement with the full-dimensional model while differing greatly compared to the plasma-free case, illustrating that we are well within the overdriven limit and that one-dimensional models (*9*) or those that neglect plasma back-reaction (*14*) will overestimate the ionization fraction by up to two orders of magnitude. The ionization fraction is only weakly dependent on both gas pressure and gas species, illustrating that saturation effects are critically important under our experimental conditions. To demonstrate that this overdriven regime results in the optimal generation, we varied the driving laser intensity. Decreasing the driving intensity either through looser focusing or by applying an aperture to the beam was found to result in such a rapid, monotonic decrease in the harmonic flux that the signal dropped below detectable levels before reaching nominal on-target intensities consistent with single-atom cutoffs in the water window (section S6 and fig. S7). Our results thus suggest that, for high photon energies and long-wavelength drivers, harmonic generation in the overdriven limit (*26*, *27*) is considerably more efficient than the usual adiabatic case where the plasma-induced evolution can be neglected (*21*).

### Temporal and spatial characteristics

To show that isolated x-ray pulses are produced even from the strongly overdriven plasma, we measured the CEP dependence of the harmonic spectrum. This dependence is shown at the two most extreme spectral regions of the water window (284 and 540 eV) in Fig. 2 (B and E), with generating conditions corresponding to the indicated regions in Fig. 2 (A and D). Lineouts at two CEP values, θ and θ + π/2, are shown in Fig. 2 (C and F). Clear CEP-dependent modulation is observed at both extreme edges of the water window and even beyond the oxygen K-edge. CEP-dependent modulation is a direct indicator of the subcycle temporal nature of the harmonic emission (*22*) and of the emission of attosecond pulses (*16*, *23*).

**Fig. 4:**
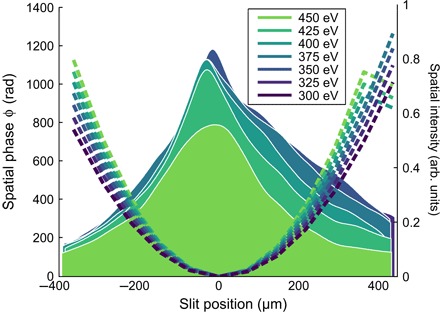
Wavefront of harmonics generated in 2 bar of helium, measured with SWORD, with spatial intensity shown as solid regions and spatial phase shown as dashed lines for seven photon energies between 300 and 450 eV. The 10-μm slit is positioned 128.5 mm upstream from the gas target.

As a test of the spatiotemporal structure, we measured the harmonic wavefronts with the Spectral Wavefront Optical Reconstruction by Diffraction (SWORD) technique (*28*). The measured wavefront for HHG in 2 bar of helium is shown in Fig. 4. The wavefront is parabolic across 150 eV of bandwidth in the water window, showing that high spatial quality harmonics can be generated even in the overdriven limit. Because of the strong spatiotemporal reshaping, it is likely impossible to achieve high-flux harmonic generation without a pristine input pulse as in our case, in which we ensure a good spatial profile for the harmonics. Our measurement allows us to determine precisely the location of the virtual harmonic source introduced by the intensity-dependent dipole phase (*21*). We find that for all harmonic orders the virtual foci coincide, with the radiation appearing to originate 8.5 ± 0.7 mm before the gas target for 2 bar of helium and 10.5 ± 0.8 mm before for 4 bar of helium. This marks a departure from harmonic generation with 800-nm lasers, where the wavefront is strongly frequency-dependent (*28*), and shows that all frequencies in the present case can be brought to a common focus.

### Propagation in the overdriven limit

**Fig. 5:**
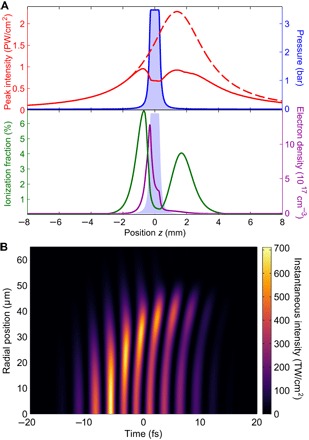
Propagation of fundamental laser. (**A**) On-axis peak intensity (red line), peak intensity neglecting ionization (red dashed line), gas density (shaded area), on-axis ionization fraction (green line), and final electron density (purple line) along the propagation direction for a backing pressure of 4 bar of helium. (**B**) Spatiotemporal structure of the fundamental laser field, on axis at the center of the gas target.

To better understand the macroscopic effects in the overdriven regime, we have simulated the laser propagation and resulting harmonic generation (see details in section S8). The model accurately predicts the ionization fraction of the medium, as shown in Fig. 3. Figure 5A shows the evolution of the 1.8-μm driving field with a backing pressure of 4 bar of helium. The laser pulse is strongly refracted and defocused by plasma generated in the tails of the gas distribution, even significantly before the inside of the target. The most apparent effect of the plasma defocusing is that it clamps the laser intensity at the gas target to around 7 × 10^14^ W/cm^2^, causing the deviation between the nominal and experimentally observed cutoff. Off-axis components, which are only weakly affected, come to focus after the target, causing the double-peak structure. Focusing after the target causes a relatively slow intensity variation through the gas, minimizing the intensity-dependent dipole phase gradient, which is the likely explanation of the global optimization of harmonic flux we observe at this focal position. Our simulations show that, without adjusting the focus, it would be necessary to decrease the intensity and harmonic cutoff to 3.5 × 10^14^ W/cm^2^ and 350 eV, respectively, to avoid significant distortions. Conversely, doubling the input energy results in only a 10% increase in the intensity at the center of the gas target. Although we consider a few-cycle laser field in which ionization is suppressed, the increasing effect of the free-electron plasma upon long-wavelength driving fields (scaling as refractive index *n* ∝ λ) means that plasma defocusing remains of critical importance (*29*).

In addition to clamping the peak intensity, the plasma response also reshapes the laser field spatiotemporally. The generation of plasma on-axis results in self-compression by defocusing the trailing portion of the pulse, increasing the relative contrast of the leading half-cycles. The effect is much smaller off-axis, leading to the crescent-shaped spatiotemporal structure seen in Fig. 5B. The on-axis pulse compression leads to the formation of a single-cycle pulse; this could be a significant effect in the generation of high-harmonic supercontinua from multicycle driving fields (*30*). Furthermore, the negative contribution of the plasma to the refractive index causes the trailing half-cycles to “catch up” to the leading ones and blueshift (*31*), which has been observed in previous works on HHG in the water window (*11*). We can further observe that a significant blueshift is limited to the weaker trailing half-cycles, which also exhibit strong radial gradients, and thus will have smaller on-axis harmonic emission than predicted by one-dimensional models (*32*). Considering only the on-axis contribution, compression leads to the generation of individual attosecond pulses, even from what would have been plateau harmonics in the unperturbed case. Although the off-axis pulse duration remains longer, the spatial gradient and cycle-to-cycle variation in intensity result in strongly varying phase-matching conditions for each half-cycle (*26*, *33*), a direct consequence of the spatiotemporal coupling.

### Transient phase matching of harmonic radiation

**Fig. 6:**
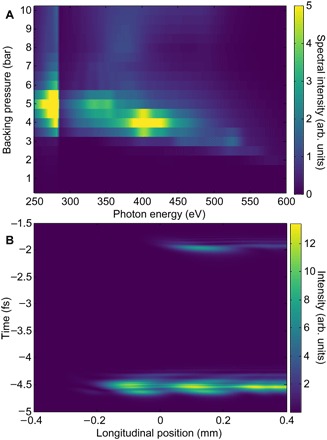
Phase matching of harmonic radiation. (**A**) Theoretical harmonic spectrum as a function of backing pressure, averaged over three values of the CEP. The plot is partially saturated to reveal high pressure and energy features. (**B**) Buildup of on-axis harmonic radiation as a function of longitudinal position inside the gas cell considering a pulse filtered for energies about 284 eV. A backing pressure of 4 bar is used, with the vacuum focus 1.4 mm behind the target and a CEP offset of 0.3**π**. The inner diameter of the target spans from −0.257 to 0.257 mm.

To obtain greater insight into how the overdriven regime results in efficient buildup, we have carried out detailed simulations of the macroscopic harmonic generation. Figure 6A shows the pressure dependence of the theoretical harmonic spectrum emitted from helium. We observe behavior similar to the experimental results (Fig. 2D), with the flux increasing with pressure toward a maximum at around 4 or 5 bar. Along with the evolution of the flux with the backing pressure, the model also reproduces the gradual decrease in the harmonic cutoff energy as the backing pressure is increased, as well as the focal position corresponding to the maximum flux for a backing pressure of 4 bar (fig. S2). The harmonic flux decreases with higher backing pressure despite the slowly varying free-electron density (Fig. 3) as the plasma generation occurs significantly upstream in the gas density profile of the target, changing the conditions for macroscopic phase matching. Our simulations additionally predict a divergence of 0.9 mrad at 284 eV from 4 bar of helium, compared to 1.1 mrad found in experiment.

We now examine the macroscopic buildup along the propagation axis. Figure 6B shows the buildup of the on-axis harmonic radiation as a function of the propagation distance inside the medium in time. We calculate the cumulative buildup as

$$S(t,z)=\int_{-\infty }^{z}D(z\prime ,t)dz\prime$$ (1)

where *S*(*t*, *z*) is the on-axis (zero transverse wave number) harmonic field at position *z*, *D*(*z*′, *t*) is the on-axis harmonic emission from the plane at *z*′, and *t* is the retarded time. Each plane has the effects of gas dispersion and absorption en route to the detector applied, so *S*(*t*, *z*) already includes subsequent propagation effects. We apply a spectral filter to the results to consider only harmonic frequencies above the carbon K-edge; harmonics are emitted up to 450 eV, and the equivalent plot in the spectral domain is shown in fig. S8. Here, we consider a 4-bar backing pressure and a CEP offset of 0.3π, but the results are representative of all simulation results. The harmonic buildup occurs very rapidly, with the attosecond burst at −5 fs going from 10 to 90% of its maximum value within 100 μm, starting around −0.2 mm, before passing through several Maker fringes of approximately 200-μm period. The secondary attosecond pulse at −2-fs phase matches starting at 0 mm and similarly goes through a rapid buildup over a distance of 100 μm and then a Maker fringe. The final emission is dominated by a single attosecond burst, which is attributable solely to macroscopic effects. The single-atom response at any given plane consists of multiple pulses, some with cutoff energies beyond the highest phase-matched photon energy, but only the phase-matched contributions survive propagation. This shows the fundamentally nonadiabatic nature of the phase matching in the overdriven regime, with the phase-matching conditions varying strongly from half-cycle to half-cycle within the generation region (*26*, *34*, *35*). One-dimensional models have shown that this can lead to very efficient generation (*32*). The on-axis ionization fraction across the 100-μm region over which phase matching is achieved is around 1%, significantly higher than the <0.1% ionization fraction predicted to result in good phase matching from previous models (*9*, *20*).

Full-dimensional models of overdriven HHG show that isolated attosecond pulses can be generated either directly in the medium (*33*) or by spatial filtering in the far field because each half-cycle burst is emitted with a different divergence (*26*, *36*). We have observed both regimes in our simulations and find that for harmonics above the carbon K-edge at 4 bar and integrated across the transverse direction, 60% of CEP values give an isolated attosecond pulse, where a pulse is deemed isolated if the second brightest pulse has less than 15% the energy of the brightest. In the remaining cases, further spectral selection of just the highest photon energies would once again result in isolated attosecond pulses. In all cases, simulations predict durations at the detector between 95 and 240 as FWHM, several times the transform limit due to the attochirp. On the basis of the agreement of simulation with experiment, we infer that, with 1:1 imaging of the virtual source plane, we could deliver a pulse with an approximately 100 as duration and a spot size of around 1 μm to experiments.

### Optimization of SXR HHG

We are now in a position to outline some general principles for the optimization of SXR HHG. SXR harmonic generation requires the use of longer-wavelength driving fields than the traditional 800 nm (*9*). As we have shown here, generation is optimized by entering the overdriven regime. While we have shown this optimization using experiment and comprehensive theory calculations, it also follows from heuristic wavelength-scaling laws. First, the magnitude of the change in the refractive index produced by the plasma |Δ*n*| depends on both wavelength and number density *N* as $|\Delta n|\propto \lambda \sqrt{N}$. Because the number density required for phase matching also increases with wavelength (*9*), significant distortion of the driving field occurs at much lower intensities for longer wavelengths. Thus, although the harmonic cutoff energy *E* scales as *E* ∝ *I*λ^2^, the wavelength cannot scale arbitrarily even for a fixed intensity without entering the overdriven regime. Second, the phase slip of the fundamental that can be tolerated in HHG scales as the ratio of the generated wavelength to the driving wavelength λ_HHG_/λ_0_, whereas the intensity dependence of the dipole phase scales as $\lambda_{0}^{3}$ (*37*). Thus longitudinal phase matching becomes increasingly difficult to achieve and will, in most realistic circumstances, be limited to relatively short longitudinal regions (*35*). Because macroscopic buildup occurs transiently and at higher intensities in the overdriven limit, it becomes increasingly favorable compared to the low-ionization limit for HHG (*9*, *21*) as the photon energies and driving wavelengths increase. Although we carried this work out at 1.8 μm, as HHG moves to even longer-wavelength driving fields, the overdriven regime will become even more important to the generation of bright SXR harmonics; some criteria for determining whether a system operates in the overdriven limit are shown in section S10. Because of the role of the plasma refractive index in this regime, it is crucial that the drive field have an excellent spatial profile, as was the case in our work, to avoid detrimental spatiotemporal coupling in the HHG dynamics. Moreover, in this regime, all geometries will be strongly affected by these wavelength scaling factors, including the capillary-based schemes where defocusing will lead to strong coupling to higher modes, which will, in turn, lead to intensity beating and dephasing.

## DISCUSSION

Within the overdriven limit, there are several routes to even brighter harmonic emission. Using shorter driving laser pulses leads to more energy in the strongest half-cycle and suppresses the ionization relative to the intensity, resulting in higher-harmonic energy and flux. We performed infrared (IR)–only simulations that showed that the intensity delivered to the target increases as the pulse duration is decreased. This occurs despite the increase in peak ionization rate. Particularly with regard to pulses of 7- to 9-fs duration at 1.8 μm, corresponding to previously reported pulse parameters (*24*), our simulations show that effective delivery of intensities supporting 800-eV harmonics is possible. We are currently limited by the length over which phase matching occurs (approximately 100 μm) and not by the absorption length (5 mm at 284 eV and 34 mm at 540 eV for 4 bar of helium). This suggests than the use of submillimeter-length targets is more appropriate than use of longer targets which increase absorption and dephasing. Improvements to the coherence length could also provide a route toward optimization of high-harmonic flux in the water window. This can be achieved through the use of gas gradient shaping, quasi–phase matching (*38*), and spatiotemporal shaping of the fundamental driving field (*39*). These techniques all take on significantly more importance for overdriven SXR harmonics, in which the harmonic flux is far from absorption limited (*40*).

In summary, we have demonstrated a new route toward the generation of bright harmonics in the SXR range by overdriving the generation medium. With laser pulses focused to intensities much higher than the critical plasma intensity, we have generated CEP-dependent harmonic cutoffs tunable from the carbon K-edge to 600 eV, almost 200 eV higher than the conventional harmonic cutoff energy, and with a photon flux in the water window an order of magnitude higher than previously reported. Measurements of the plasma density combined with comprehensive theoretical modeling show that the plasma response is the dominant contribution to the macroscopic response, clamping the intensity and reshaping the fundamental laser, limiting the maximum plasma fraction to a few percent. Provided the driving pulse has a good spatiotemporal quality, as in our experiments, this results in transient buildup of harmonics with a well-behaved spatial phase, as confirmed by SWORD measurements. Applying this newfound understanding of spatiotemporal reshaping of the driving field as the critical parameter, we outline several approaches to further optimize HHG in the SXR region. Harmonic generation in the overdriven limit is critical for the development of bright SXR attosecond sources and will become even more important as attosecond pulses move to even higher photon energies and longer-wavelength driving fields.

## MATERIALS AND METHODS

### Experimental setup

A chirped pulse amplification laser (Red Dragon, KMLabs) delivered 8 mJ, 800-nm pulses that were 30 fs in duration at a repetition rate of 1 kHz. The pulses were used to pump an optical parametric amplifier (OPA) (HE-TOPAS, Light Conversion). Pulses at the idler wavelength of 1.8 μm with a pulse energy of 1.4 mJ and a duration of ≈30 fs were coupled into a 400-μm inner diameter, 1-m-long hollow capillary for pulse compression. The input of the capillary was held at vacuum pressure, and the exit was filled with 0.4 bar of argon. Using the anomalous dispersion of silica, the output pulses were compressed to a duration of 12 fs at the experiment (*15*). The pulses were characterized spatiotemporally using SEA-F-SPIDER, which allowed us to ensure the high spatiotemporal quality (*24*) that is required in the overdriven regime, where plasma effects from ionization dominate the field propagation dynamics. The high-energy, few-cycle pulses were then sent to the harmonic generation setup, with an on-target pulse energy of 550 μJ, corresponding to 750 μJ of output from the fiber. The CEP of the idler was passively stabilized via the design of the OPA; slow drifts were corrected using a piezo mirror delay inside the OPA controlled by a 2f-to-3f interferometer after the fiber, resulting in a stability of 300 ± 50–mrad root mean square (single shot).

The CEP-stable, two-cycle pulses were focused using a 20-cm CaF_2_ lens into an 820-μm outer diameter, 514-μm inner diameter needle filled with up to 16 bar of helium. Because of the low dispersion of CaF_2_ across the 1600- to 2100-nm spectral range, we calculated that the chromatic aberration of the lens was not significant in these measurements, and the use of looser than f/10 focusing suggested negligible spherical aberration; other cylindrically symmetric aberrations can also reasonably be expected to have minimal impact due to the dominance of the plasma lensing. The 40-μm FWHM focus was scanned to drill holes of 210-μm diameter into the target, which the beam could pass through unimpeded at any focal position. Two stages of differential pumping surrounded the target and maintained an ambient pressure of less than 10 mbar around the target and less than 10^−2^ mbar outside the differential pumping. The harmonics and fundamental propagated through a 200-nm-thick aluminum filter, which removed the fundamental. Harmonics propagated through a 650-μm slit onto a variable line spacing grating with nominal groove spacing of 2400 lines/mm (Shimadzu 30-003) and were dispersed onto an x-ray CCD camera (Andor Newton SO). The role of the slit was to ensure that only a limited region of the grating is illuminated to avoid significant aberrations that are detrimental to the spectral resolution. The photon-counting ability of the x-ray CCD allowed us to extract the absolute harmonic flux.

### SWORD measurement

A 10-μm slit was introduced into the harmonic beam oriented perpendicular to the spatial axis of our imaging spectrometer (Fig. 1D) and scanned across the harmonic beam in steps of approximately 150 μm. Eleven positions were recorded in total. The transmitted light was sent into the imaging spectrometer. The position of the central order relative to the slit position encoded the local phase gradient; when spectrally resolved, we were able to reconstruct the wavefront of the harmonics for each photon energy (see the Supplementary Materials for details).

### Spectral phase measurement of ionization fraction

Weak pre- and postpulses at a central wavelength of 800 nm were co-propagated through the target along with the generating pulse, separated from the harmonics by an annular mirror (Fig. 1C) and sent into a spectrometer. The spectral interference of the two pulses encoded the total ionization fraction of the medium (see the Supplementary Materials for details).

### Simulations

Our model treats the IR pulse by solving the forward Maxwell equation, explicitly including all spatial dimensions, as well as ionization and the χ^3^ nonlinearity with self-steepening. We treated the target geometry realistically, through interferometric determination of the density-length product and comparisons to literature density distributions. The harmonic radiation was calculated via the strong-field approximation at each plane using the spatiotemporal profile of the 1800-nm pulse from the propagation simulations (*37*). The radiation from each plane was coherently propagated, including dispersion, absorption, and diffraction, to the detector plane and integrated to produce the total macroscopic field. Further details are shown in the Supplementary Materials.

### Statistical analysis

The harmonic flux was determined from the camera efficiency, grating diffraction efficiency, spectrometer angular acceptance, filter transmission, and carbon absorption. The error on the total flux was calculated through the usual error propagation methods from the errors from each individual factor, taken to be negligible for the camera, 13% on the grating efficiency, 7% on the spectrometer angular acceptance, 10% on the filter thickness, and 25% on the thickness of the carbon absorption layer (see details on these values in the Supplementary Materials). These factors gave an error of 26% on the final harmonic flux.

Errors in the virtual source positions determined with SWORD are residuals from the least-squares fit made to the reconstructed phase profile. Errors in the ionization fraction measurements were taken as the extreme values over three measurements.

## Supplementary Material

http://advances.sciencemag.org/cgi/content/full/4/5/eaar3761/DC1

## SUPPLEMENTARY MATERIALS

Supplementary material for this article is available at http://advances.sciencemag.org/cgi/content/full/4/5/eaar3761/DC1 section S1. Role of filamentation section S2. Focal position scan section S3. Harmonic flux section S4. Oxygen K-edge XANES section S5. Spectral phase interferometry of plasma density section S6. Thirty-centimeter focusing results section S7. Spatial wavefront characterization section S8. Simulation methods section S9. Harmonic buildup section S10. Overdriven limit fig. S1. Gas flow from thin needle target. fig. S2. Harmonic focal scans. fig. S3. Oxygen K-edge spectroscopy. fig. S4. Spectral interferometer for plasma characterization. fig. S5. Plasma-induced phase shifts. fig. S6. Linear-scale plasma phase shift. fig. S7. Harmonics generated with looser focusing. fig. S8. Buildup of harmonic flux. fig. S9. Buildup of harmonic flux. table S1. Harmonic flux in the water window. References (*41*–*54*)
