# Supplementary material for: High-flux soft x-ray harmonic generation from ionization-shaped few-cycle laser pulses
Source: Sci Adv. 2018 May 11;4(5):eaar3761. doi: 10.1126/sciadv.aar3761 (PMC5947981; doi:10.1126/sciadv.aar3761)
Supplement: http://advances.sciencemag.org/cgi/content/full/4/5/eaar3761/DC1 [file supp_4_5_eaar3761__index.html]

Science Advances | Science Advances

## Supplementary Materials

**This PDF file includes:**

- section S1. Role of filamentation
- section S2. Focal position scan
- section S3. Harmonic flux
- section S4. Oxygen K-edge XANES
- section S5. Spectral phase interferometry of plasma density
- section S6. Thirty-centimeter focusing results
- section S7. Spatial wavefront characterization
- section S8. Simulation methods
- section S9. Harmonic buildup
- section S10. Overdriven limit
- fig. S1. Gas flow from thin needle target.
- fig. S2. Harmonic focal scans.
- fig. S3. Oxygen K-edge spectroscopy.
- fig. S4. Spectral interferometer for plasma characterization.
- fig. S5. Plasma-induced phase shifts.
- fig. S6. Linear-scale plasma phase shift in neon (red) and helium (blue).
- fig. S7. Harmonics generated with looser focusing.
- fig. S8. Buildup of harmonic flux.
- fig. S9. Buildup of harmonic flux.
- table S1. Harmonic flux in the water window.
- References (*41–54*)

Download PDF

**Files in this Data Supplement:**

- Adobe PDF - aar3761\_SM.pdf
