# Supplementary material for: High-flux soft x-ray harmonic generation from ionization-shaped few-cycle laser pulses
Source: Sci Adv. 2018 May 11;4(5):eaar3761. doi: 10.1126/sciadv.aar3761 (PMC5947981; doi:10.1126/sciadv.aar3761)
Supplement: http://advances.sciencemag.org/cgi/content/full/4/5/eaar3761/DC1 [file aar3761_SM.pdf]

Science Advances

AAAS

advances.sciencemag.org/cgi/content/full/4/5/eaar3761/DC1

Supplementary Materials for

High-flux soft x-ray harmonic generation from ionization-shaped few-cycle laser pulses

Allan S. Johnson, Dane R. Austin, David A. Wood, Christian Brahms, Andrew Gregory, Konstantin B. Holzner, Sebastian Jarosch, Esben W. Larsen, Susan Parker, Christian S. Strüber, Peng Ye, John W. G. Tisch, Jon P. Marangos

Published 11 May 2018, *Sci. Adv.* **4**, eaar3761 (2018)
DOI: 10.1126/sciadv.aar3761

This PDF file includes:

- section S1. Role of filamentation
- section S2. Focal position scan
- section S3. Harmonic flux
- section S4. Oxygen K-edge XANES
- section S5. Spectral phase interferometry of plasma density
- section S6. Thirty-centimeter focusing results
- section S7. Spatial wavefront characterization
- section S8. Simulation methods
- section S9. Harmonic buildup
- section S10. Overdriven limit
- fig. S1. Gas flow from thin needle target.
- fig. S2. Harmonic focal scans.
- fig. S3. Oxygen K-edge spectroscopy.
- fig. S4. Spectral interferometer for plasma characterization.
- fig. S5. Plasma-induced phase shifts.
- fig. S6. Linear-scale plasma phase shift in neon (red) and helium (blue).
- fig. S7. Harmonics generated with looser focusing.
- fig. S8. Buildup of harmonic flux.
- fig. S9. Buildup of harmonic flux.
- table S1. Harmonic flux in the water window.
- References (*41–54*)

S1 Role of Filamentation

We observed a bright gas plume, extending several millimeters either side of the gas needle target. However in at least one case (shown in Fig. S1), the plume was emitted at an angle to the laser propagation direction due to slight misalignment of the laser drilled holes. This indicates that the plume is not a filament, as along the laser propagation direction plasma emission is restricted to less than 1 mm either side of the target. Observation of such plumes cannot be used as evidence of filamentation, and our observation shows that in this regime filamentation is not important for the efficient generation of SXR harmonics.

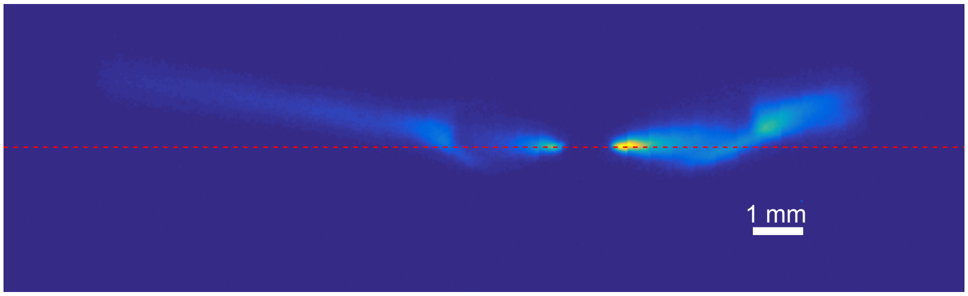

fig. S1. Gas flow from thin needle target. Photo of gas plume from above. Due to extreme contrast only the plume and focus themselves are visible, and not the target or chambers. The dark zone is the center is caused by the target obscuring the light. Laser propagation direction is indicated by the dashed red line.

S2 Focal Position Scan

Figure S2 shows the experimental and theoretical harmonic radiation emitted from a focal position scan from 4 bar of helium. The experimental data was taken averaged over all CEP. The harmonic flux is optimized focusing after the target, in direct contrast to predictions from the weak ionization limit (*21*), and is optimized simultaneously for all photon energies. The theoretical harmonic spectra is averaged over two carrier-envelope phases that differ by $\pi/2$. The agreement between theory and experiment is good, in both harmonic cut-off (around 550 eV) and the range of focal positions over which harmonics are efficiently generated. The absolute offset between the experimental and theoretical maxima (0.5 mm) is attributable to the uncertainty in the absolute focal position of the experimental harmonics. This focal position is measured by observing the location of the plasma generated when the chamber is filled with a low backing pressure (20 mbar) of helium, but simulations show that nonlinear effects can shift the focus forward by around 0.5 mm even in this case. Because of this uncertainty, we performed all simulations at z=1.389 mm, the position of maximum flux in our focal position scan.

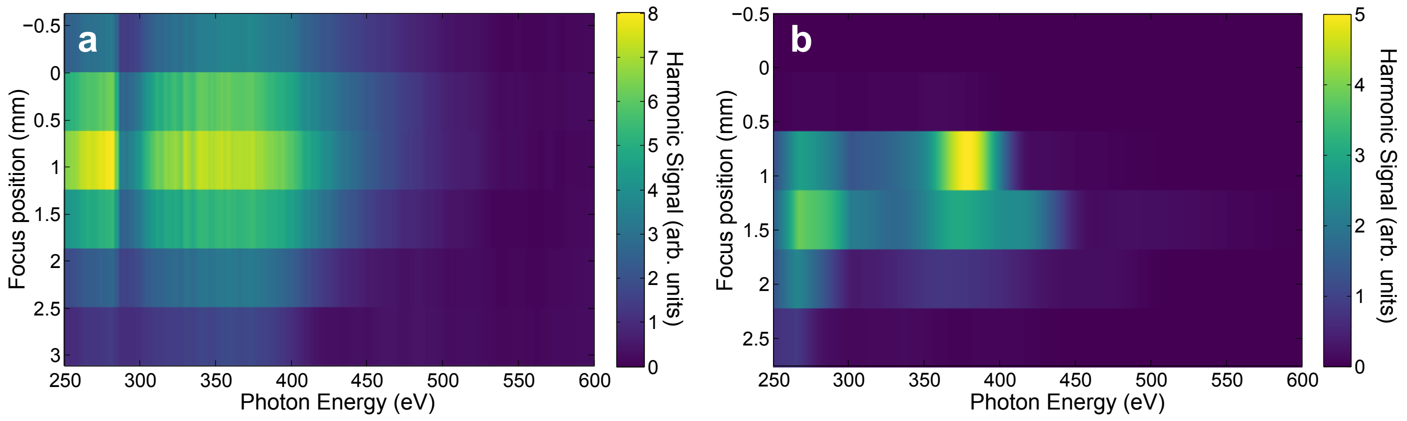

fig. S2. Harmonic focal scans. Harmonic spectrum from 4 bar of helium as a function of focal position. Negative numbers correspond to the vacuum focus before the center of the needle. **a** Experimental data. **b** Simulation results, averaged over two carrier-envelope phases that differ by $\pi/2$.

S3 Harmonic Flux

Flux determination. The factors affecting the harmonic flux calculation are discussed here. The quantum efficiency of the camera is provided by the manufacturer, separately as the photon absorption probability and the electrons generated per photon. The efficiency of the grating was also available. The filter transmission is estimated using the manufacturer specified thickness and the atomic scattering factors (*41*). The effect of absorption by carbon contamination is modelled as an effective atomic carbon filter, whose thickness is adjusted to match the sharp drop in signal at the K edge. This approximation is well justified far from resonances, but results in artefacts at the π^* resonance, for which reason we quote fluxes away from this region only (i.e. at 300 eV as a proxy for the carbon K edge). The angular acceptance of the spectrometer is taken into account by combining the known slit width (650 µm) together with the beam profile in the transverse direction. The loss at the slit can be determined independently for each measurement and is robust to changes in the beam divergence.

Ref.	Gas	300 eV	400 eV	540 eV	Total WW Flux
This work	Ne	$(3.0 \pm 0.8) \times 10^6$	$(5.6 \pm 1.4) \times 10^4$	0	71 ± 18 pJ (1 kHz)<br>$(1.4 \pm 0.4) \times 10^9$ photons/s
This work	He	$(1.6 \pm 0.4) \times 10^5$	$(4.5 \pm 1.2) \times 10^4$	$(1.1 \pm 0.3) \times 10^3$	7.7 ± 2.0 pJ (1 kHz)<br>$(1.4 \pm 0.4) \times 10^8$ photons/s
(*14*)	Ne	-	-	-	2.9 ± 0.1 pJ (1 kHz)<br>$(7.3 \pm 0.1) \times 10^7$ photons/s
(*14*)	He	-	-	-	0.9 ± 0.2 pJ (1 kHz)
(*16*)	Ne	-	-	-	$> 1 \times 10^8$ photons/s
(*20*)	He	-	-	-	6×10^7 photons/s

table S1. Harmonic flux in the water window. Comparison of flux generated at various edges and integrated across the water window. Flux is quoted as photons/s/1% bandwidth for the edges, and as either photons/s or energy per pulse for the total water window. Flux is measured at 300 eV as a proxy for the carbon K edge to avoid possible artefacts from contamination.

The uncertainty incorporates the uncertainties of the filter transmission, the thickness of the carbon layer, the efficiency of the grating, and the slit width. The transmission of the aluminium filter is taken to be accurate to 10%, a conservative estimate (*42*). The thickness of the carbon filter is fit to the data; as we have observed that over our experiments the effective thickness increased from 100 nm to 150 nm, we have taken the uncertainty for any given scan to be 25 nm. The uncertainty on the reflectivity of the grating was taken to be 13% by considering the error of a typical grating characterization measurement (*43*). Finally, the accuracy of our measurement of the slit width was 50 µm, determined by repeated measurements of the slit width. The combination of these factors gives a 26% error in the final harmonic flux. The error on the XUV camera's manufacturer provided quantum efficiency is taken to be negligible here, but we have observed thin-films deposited onto the face of the XUV camera that could well negatively impact the camera sensitivity. Depending on the severity of this uncontrolled factor, the flux could be significantly higher than quoted here.

S4 Oxygen *K* Edge XANES

Figure S3 shows the XANES spectrum of a 1 μm thick film of biaxially-oriented polyethylene terephthalate (BoPET) with XANES features from the literature indicated (*44*). Harmonics were generated in 2 bar of He, with a cutoff extending beyond 600 eV. Alternating 5 min exposures were taken with and without the BoPET for a total measurement time of 3 hours. The source was found to be extremely stable over this time frame. Improvements in the collection efficiency should decrease the acquisition time by two orders of magnitude, easily allowing for time-resolved studies. These improvements include the use of thinner filters (here limited by the presence of pinholes), removal of carbon contamination by ozone cleaning, and the use of a collection optic such as a toroidal mirror in order to optimally match the beam to the correct aperture on the grating to minimize the aberration without recourse to a flux limiting slit.

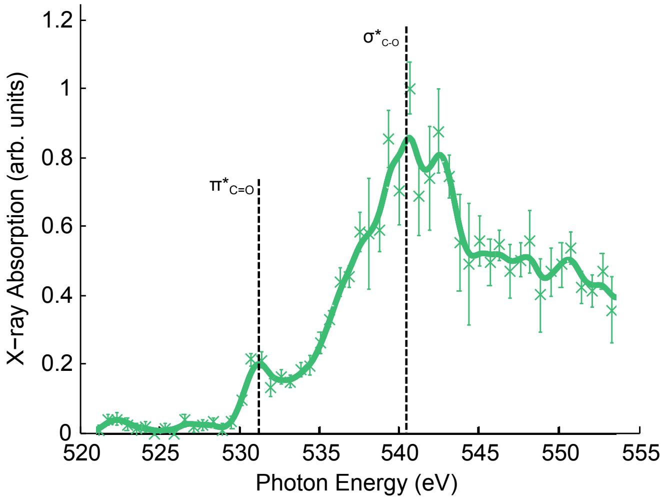

fig. S3. Oxygen *K*-edge spectroscopy. XANES spectrum of BoPET film measured with high harmonic radiation. Error bars represent the standard error about the mean over all acquisitions. The solid line shows a moving average over two points as a guide to the eye.

S5 Spectral Phase Interferometry of Plasma Density

Optical Setup. We measure the final electron density of the plasma generated by the 1800 nm driving laser pulse via spectral phase interferometry (SPI). The apparatus is shown in Fig. S4. A small portion of the 800 nm CPA used to pump the OPA is split off prior to the OPA. The delayed pulse is then passed through a compact Mach-Zender interferometer. A controllable delay in one arm is used to produce a pair of pulses at the output with a separation of ≈ 500 fs. These pulses are then combined collinearly with the 1800 nm pulse by reflection off the Brewster angled compression wedges. The 800 nm pulses are low power to ensure that there is no disruption of the harmonic generation. Using the overall delay of the 800 nm arm, the delay of the pulses is adjusted until one 800 nm pulse leads the 1800 nm pulse in time while the other trails. All three pulses then pass through the target and are picked off by an annular mirror, while the much lower divergence harmonics pass through unaffected. By examining the harmonics we observed the weak 800 nm radiation has no effect upon the harmonic generation or 1800 nm pulse propagation. The pulses are recollimated and pass through a shortpass filter to remove the strong 1800 nm beam before being focused into an NIR spectrometer.

Stability and Calibration. Although the 800 nm pulses were separated from the 1800 nm over 10 m before the recombination, the requirements on the temporal stability of the interferometer are very weak, as all that is required is that one pulse remains before the 1800 nm and the other afterwards with fixed timing between the two 800 nm pulses. The time delay between the two 800 nm pulses was determined from the spectral interferometry fringes; the temporal overlap with the 1800 nm was found by scanning the global time delay and finding the region where the spectral interferometry showed a blue shift of the fringes. When the delay was adjusted so both 800 nm pulses were before or after the 1800 nm, the shift was lost as both pulses experience the same delay. As the 800 nm pulses had a bandwidth of $\approx$50 nm, we can differentiate between a shift of below π radians and a larger shift, allowing us to state definitively the shift was below π radians, as shown in Fig. S5.

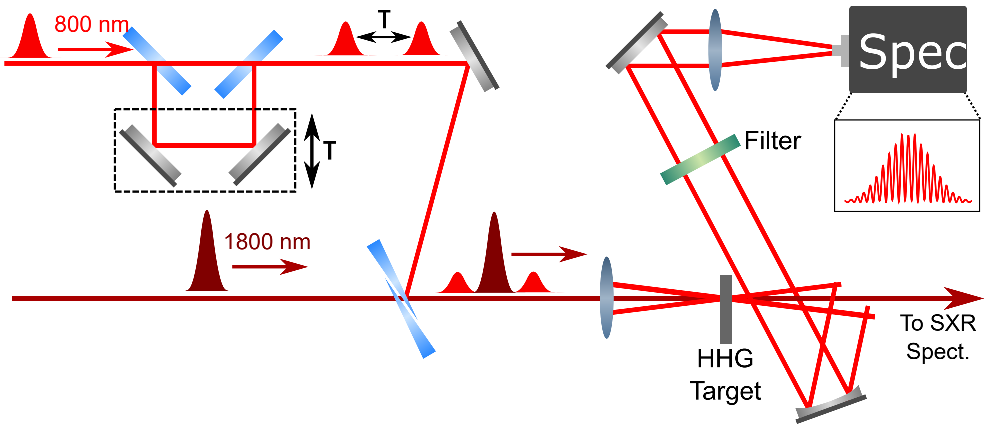

fig. S4. Spectral interferometer for plasma characterization. Schematic of scheme for the characterization of plasma density. Two identical weak 800 nm pulses with controllable time delay are created and combined colinearly with the fundamental pulse such that one leads and one trails the strong mid-IR pulse. After harmonic generation the optical pulses are separated from the harmonics and the mid-IR filtered. The 800 nm pulses are then sent into a spectrometer, where the spectral interferogram encodes the effect of the plasma.

Theoretical Curves. Theoretical predictions for the plasma phase shift were calculated by propagating the 1800 nm pulse to build up realistic plasma densities, then calculating the local change in $k(\vec{r})$ at 800 nm. The total phase shift was calculated as

$$\phi_{SI} = \int dz \frac{\int dr\ 2\pi r|E(r,z)|\Delta k(r,z)}{\int dr\ 2\pi r|E(r,z)|} \tag{1}$$

where the contribution from each volume element was weighted by the electric field amplitude of an 800 nm Gaussian $E(r,z)$ of beam waist 64 µm with the longitudinal position of the focus 2.54 mm upstream of the 1800 nm focus. This shift is due the chromatic aberration of the CaF_2 focusing lens. The theoretical curves are only weakly dependent upon the beam waist or focus position, which introduce a small overall scaling factor to the curve. The data and full theoretical curves are shown in Fig. S6 on a linear scale; the theoretical curves ignoring plasma defocusing are over an order of magnitude higher (see Fig. 3 of main text).

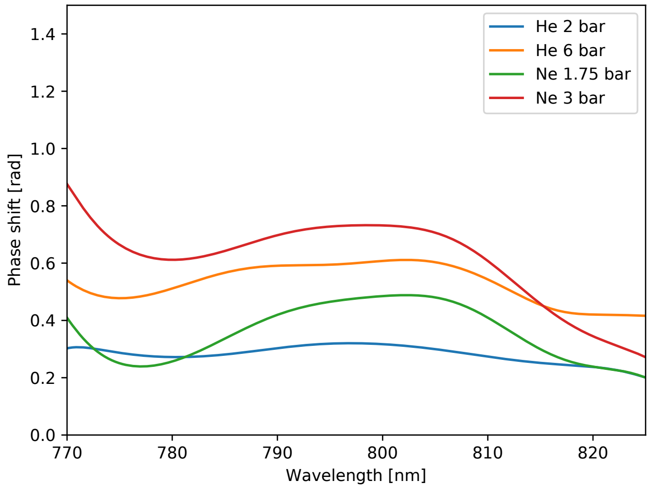

fig. S5. Plasma induced phase-shifts. Phase shifts in neon and helium resolved across the bandwidth of the 800 nm pulses.

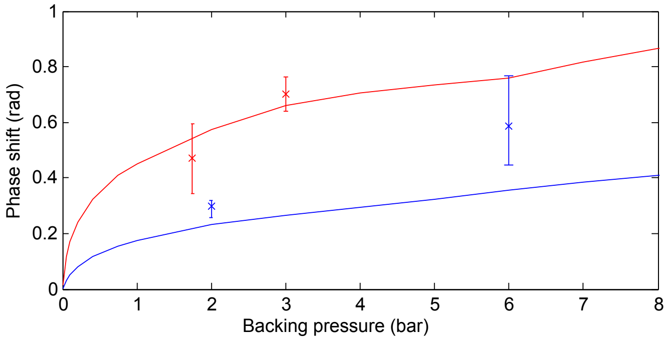

fig. S6.Linear-scale plasma phase shift in neon (red) and helium (blue). Experimental data shown as Xs, while the curve is calculated using the full dimensional simulations. Error bars represent extreme values over three acquisitions.

S6 30 cm Focusing Results

Water window high harmonic spectra were also generated using a 30 cm focal length spherical mirror as the focusing element (*15*), instead of the 20 cm CaF_2 lens used in the results presented elsewhere in this work.

Harmonics were obtained in both He and Ne, with scans over pressure, CEP, and focal position performed. Figure S7a shows the result of a backing pressure scan in neon. The overall spectral shape, dependence on pressure, and highest cutoff are all extremely similar to the generation using a 20 cm optic (Fig. 2d in main text). This suggests we are still within the same overdriven regime, and similar behaviour was also seen with respect to focal position for the two focusing lengths. However, the overall flux here is approximately half that of the tighter focusing case, and the generation was much more sensitive to the input energy.

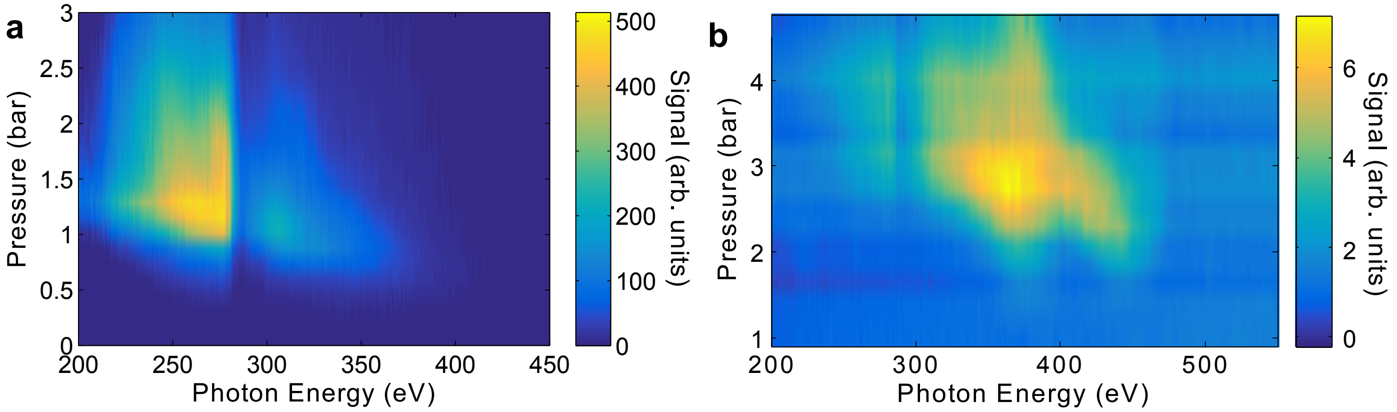

fig. S7. Harmonics generated with looser focusing. **a** High harmonic spectrum of neon as a function of backing pressure generated using a 30 cm focal length mirror as the focusing optic. **b** High harmonic spectrum of helium for a fixed CEP as a function of backing pressure generated using a 30 cm focal length mirror as the focusing optics.

In helium the generation was much more strongly affected by the change in focusing. Figure S7b shows the pressure dependence of the harmonics from helium at a fixed CEP. The flux within the water window is maximized at a much lower pressure than with the tighter focusing, and the overall flux and cutoff are considerably lower, by a factor of two to three and 150 eV, respectively. Decreasing the input energy by closing an iris almost immediately reduced the harmonic signal to below observable levels. In general HHG in He with the looser focusing condition was found to be extremely challenging and time-consuming, and

generally inferior to HHG with the 20 cm focusing optic.

S7 Spatial Wavefront Characterization

Principle and Setup. We measured the photon energy dependent spatial wavefront of the harmonics using the Spectral Wavefront Optical Reconstruction by Diffraction (SWORD) technique (*45*). A 10 µm slit is introduced into the harmonic beam and scanned across it along the imaging axis of the spectrometer. The spectrally resolved diffraction pattern is measured in the far field; the centroid of the diffraction pattern follows the direction of the phasefront of the beam at the position of the slit. Thus SWORD forms an analogue of a Shack-Hartmann sensor, replacing the lens array with a diffractive aperture. In the Fraunhofer diffraction limit, the spatial phase can be reconstructed as

$$\phi(x_n) = \frac{2\pi}{\lambda} \sum_{i=0}^{n} \frac{s_i - x_i}{d} \Delta s \tag{2}$$

where λ is the wavelength, s_i is the location of the diffraction pattern centroid in the far field, x_i is the slit position in the near field, Δs is the sample spacing of the slit, and d is the distance between the detector and the slit (*45*). We position the slit approximately 128.5 mm from the target to avoid burning, which leads to a slit to detector distance of $d = 1$ m. At this distance a slit of 40 µm could be used while still remaining in the Fraunhofer limit for 2 nm wavelength light; however we opt to use a 10 µm slit in order to ensure that the $m = \pm 1$ diffraction orders are resolvable on our detector, as an independent verification of the regime. We measure the diffraction at 12 points across the beam, taken $\approx$ 70 µm apart.

Measurements. Because of the extremely low throughput of the slit combined with the losses in the spectrometer, acquisitions of 5 min at each point are required in neon and of 10 min in helium, leading to scans of well over an hour. The intensity distributions obtained are very noisy, thus we complement the spatial phase SWORD measurement with a spatial amplitude measurement by removing the slit and matching the two planes through the phase measurement. The recovered phase is very stable and compared to a quadratic fit deviates by less than 1% at any given point. We are limited to measurements below photon energies of 450 eV due to

losses from the 10 µm scanning slit. Because the measured phase spans over one thousand radians, even this small residual error is sufficient to prevent reconstruction of the focus. As the harmonic wavelength decreases, the stability requirements for a successful SWORD measurement commensurately increase. For resolution of the exact harmonic wavefront in the soft X-ray spectral region, shearing interferometry measurements may prove more robust (*46*). Error bars on the virtual source position are inferred from the residuals in the least squares fit to the reconstructed phase profiles.

S8 Simulation Methods

Numerical Model. The laser propagates according to the forward Maxwell equation (*47, 48*) which includes dispersion, paraxial diffraction, the third-order nonlinearity $\chi^{(3)}$ (including self-steepening), plasma dephasing, and absorption (*49*). The model is three dimensional with cylindrical symmetry. Instantaneous ionization rates are calculated using the ADK formula (*50*). We numerically integrate using a preconditioned Runge-Kutta method (*51*), with adaptive step-sizing. Dispersion and diffraction are applied in the frequency-wavenumber $(\omega, k_\perp)$ domain, whilst nonlinear effects are applied in the time-space (t, r) domain. The two domains are related by the discrete Fourier transform and the quasi-discrete Hankel transform (*52*), respectively. Results in the time domain are calculated and reported in a co-moving frame with fundamental propagation in the vacuum case, thus negative times correspond to harmonic emission before the peak of the (vacuum) driving pulse. For initial conditions we use spatio-temporally Gaussian pulses matching the measured pulse parameters.

For harmonic simulations, the simulated laser field is resampled to a finer spatio-temporal grid, and the single-atom HHG dipole computed at every point using the strong field approximation (*53*) with stationary phase approximations used for the momentum and birth time integrals. Temporal apodization is used to remove the contribution of the long trajectories and reduce computation time. Because of the intensity-dependent dipole phase, long trajectories are expected to be weaker and emitted with higher divergence than the short trajectories, and thus make a minimal contribution to the macroscopic response. This approach is further justified by the good agreement between theory and experiment.The macroscopic field is then obtained by spatially

integrating the HHG single-atom dipoles, taking into account paraxial diffraction, the local gas density, and absorption and dispersion by the neutral gas.

Realistic Density Profiles. Our gas target consists of a 820 μm outer diameter, 514 μm inner diameter needle inside differential pumping. We model this target as a region of constant density 514 μm across, with the flow through the laser drilled holes modelled as a decaying Lorentzian leading asymptotically towards a low pressure background, fixed to match measurements of the ambient pressure inside the differential pumping. We use a Lorentzian form for the gas jet as it is flux preserving, and thus captures the asymptotic behaviour away from the target. The width of the Lorentzian is chosen to agree with decay rates calculated using Monte Carlo simulations of gas flow through short tubes into vacuum (*54*); this width is only weakly dependent upon the backing pressure, and so we fix the half-maximum point at 105 μm. We adjust the peak pressure inside the constant region of high pressure to match the total integrated density to interferometry measurements. Fitting the inner density to be 0.875 times the backing pressure, we obtain agreement better than 1.7% between the model integrated density and the interferometry measurements for backing pressure from 1 bar to 10 bar.

S9 Harmonic Buildup

Figure 6b in the main text shows the buildup of the harmonic radiation in time as a function of longitudinal position. Figure S8 shows the buildup in the spectral domain. In the spectral domain we see immediately the broadband nature of the buildup. Maker fringes are readily apparent across almost the entire harmonic bandwidth, as well as the weak harmonic structure resulting from the secondary pulse. The buildup in the time domain is dominated by a strong pulse at -5 fs and a weaker pulse at -2 fs. Figure S9 shows the buildup of each of those pulses as a function of longitudinal position integrated over the pulse duration, integrated over all photon energies higher than 284 eV. The Maker fringes in each pulse are clearly visible, as well as the initial rapid buildup.

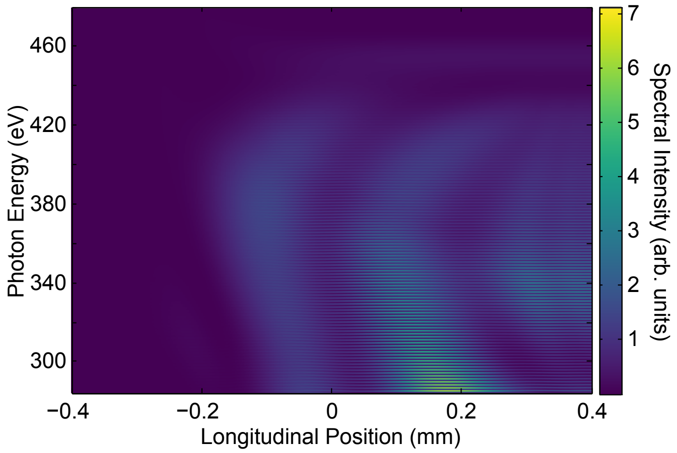

fig. S8.Buildup of harmonic flux. Buildup of the spectral harmonic flux in 4 bar of helium as a function of the propagation distance, with laser focused 1.4 mm after the gas target and a CEP of 0.3π.

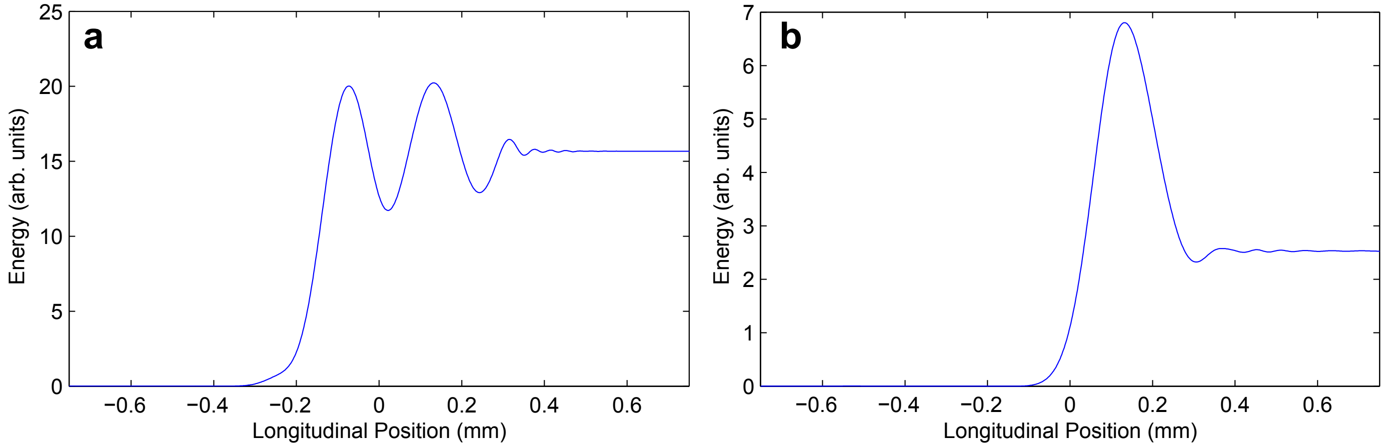

fig. S9.Buildup of harmonic flux. Buildup of the two significant attosecond pulses in 4 bar of helium as a function of the propagation distance, with laser focused 1.4 mm after the gas target and a CEP of 0.3π. **a** Flux of the brightest pulse at -5 fs. The flux builds up over 102 μm and goes through Maker fringes of approximately 200 μm periodicity. **b** Flux of the secondary pulse at -2 fs. The flux builds up up rapidly before deconstructive interference decreases it.

S10 Overdriven Limit

The overdriven regime is characterized by strong spatio-temporal reshaping of the laser pulse by laser induced ionization over a short spatial extent. As such, it is intrinsically linked to nonlinear and non-perturbative dynamics, making it difficult to treat analytically. Determining whether a system will be in the overdriven limit *a priori* from simple pulse inputs is thus a challenging prospect, but here we present some non-exhaustive criteria. There are some experimental methods by which the overdriven regime can be identified: ionization fraction is strongly linked to this value, but does not directly report it. For instance, a much higher ionization fraction is required to enter the regime at short wavelengths than long wavelengths. One method would be to perform pressure scans and examine the variation of the ionization fraction as described in section S4; the saturation effect shown is a clear indicator of strong pulse reshaping. An alternative method would be spatio-temporal characterization of the driving pulse after the medium where the back reaction of the medium on the pulse would be clear.

Although a quantitative figure of merit is difficult to define, it is reasonable to consider that the overdriven regime is entered for an 1800 nm pulse if the ionization defocusing in a thin medium clamps the peak intensity achieved to less than 70% of the vacuum propagation case. We find the fraction of peak intensity achieved can be approximated using the following empirical formula obtained from our numerical results:

$$F(I,\lambda,\omega) = \left[1.1 - \frac{I}{3.8I_0}\right]\left[1 - \frac{I}{2.5I_0}\left(\sqrt{\frac{\lambda}{\lambda_0}} - 1\right)\right]\left[\frac{10.6}{10}\left(1 + \exp[-A(\frac{w}{w_0} + B)]\right)^{-1}\right]$$

$$A = 2.8 - 20.3 \times \exp(-4.5\frac{I}{I_0})$$

$$B = \frac{1.4}{10} - 38.0 \times \exp(-6.3\frac{I}{I_0})$$

where F is the fractional intensity, I is the intensity, λ is the wavelength, w is the $1/e^2$ beam waist, and $I_0 = 1 \times 15$ W/cm^2, $w = 35$ µm, and $\lambda_0 = 1.8$ µm. This relationship is valid when varying λ and w while keeping the peak intensity and pulse duration of two cycles fixed, and at a backing pressure of 4 bar; the pressure scaling of F goes as approximately as P^{-1}, but the exact scaling depends strongly upon the other

parameters. A value of F less than 0.7 indicates the system is overdriven. This equation was found to function well around the regime of most interest, the transition to the overdriven regime between 0.6 to 1.5 PW/cm^2, and for wavelengths from 800 nm to 3 μm.
